# Making Expert Decisions Easier to Fathom: On the Explainability of Visual Object Recognition Expertise

**DOI:** 10.3389/fnins.2018.00670

**Published:** 2018-10-12

**Authors:** Jay Hegdé, Evgeniy Bart

**Affiliations:** ^1^Department of Neuroscience and Regenerative Medicine, James and Jean Culver Vision Discovery Institute, The Graduate School, Augusta University, Augusta, GA, United States; ^2^Department of Ophthalmology, Medical College of Georgia, Augusta University, Augusta, GA, United States; ^3^Palo Alto Research Center, Palo Alto CA, United States

**Keywords:** classification, weakly guided learning, machine learning, objective explainability, perceptual learning, subjective explainability

## Abstract

In everyday life, we rely on human experts to make a variety of complex decisions, such as medical diagnoses. These decisions are typically made through some form of weakly guided learning, a form of learning in which decision expertise is gained through labeled examples rather than explicit instructions. Expert decisions can significantly affect people other than the decision-maker (for example, teammates, clients, or patients), but may seem cryptic and mysterious to them. It is therefore desirable for the decision-maker to explain the rationale behind these decisions to others. This, however, can be difficult to do. Often, the expert has a “gut feeling” for what the correct decision is, but may have difficulty giving an objective set of criteria for arriving at it. Explainability of human expert decisions, i.e., the extent to which experts can make their decisions understandable to others, has not been studied systematically. Here, we characterize the explainability of human decision-making, using binary categorical decisions about visual objects as an illustrative example. We trained a group of “expert” subjects to categorize novel, naturalistic 3-D objects called “digital embryos” into one of two hitherto unknown categories, using a weakly guided learning paradigm. We then asked the expert subjects to provide a written explanation for each binary decision they made. These experiments generated several intriguing findings. First, the expert’s explanations modestly improve the categorization performance of naïve users (paired *t*-tests, *p* < 0.05). Second, this improvement differed significantly between explanations. In particular, explanations that pointed to a spatially localized region of the object improved the user’s performance much better than explanations that referred to global features. Third, neither experts nor naïve subjects were able to reliably predict the degree of improvement for a given explanation. Finally, significant bias effects were observed, where naïve subjects rated an explanation significantly higher when told it comes from an expert user, compared to the rating of the same explanation when told it comes from another non-expert, suggesting a variant of the Asch conformity effect. Together, our results characterize, for the first time, the various issues, both methodological and conceptual, underlying the explainability of human decisions.

## Introduction

One of the great successes of machine learning has been that intelligent machines can now accomplish highly complex tasks that once required highly trained, highly skilled human experts (Seger, 1994; LeCun et al., 2015; Goodfellow et al., 2016; Kim et al., 2016; Li et al., 2016; Hegdé and Bart, 2017). To cite but one instance, ‘expert’ machines can accurately predict which loan applicants are likely to pay back the loan, and which applicants are likely to default. Machine learning deals with instances of such astonishing feats of decision-making under real-world situations (Goodfellow et al., 2016; Kim et al., 2016).

Quite recently, however, a problem associated with these success stories has come to the fore: the process by which the machine arrived at the decision is abstract and complex enough that it can be often impossible to fathom how the machine arrived at the given decision. This makes it harder for the human ‘clients’ of these ‘server’ machines to have confidence in the server’s decision and to rely on it (de Visser, 2012; Ribeiro et al., 2016c). The extent to which a machine’s decision can be accounted for has come to be referred to as the ‘explainability’ of the decision (Einstein, 1985; Dale et al., 2010; Van Belle et al., 2016).

Explainability issues are especially common in a style of machine learning called deep learning. In deep learning, a multi-layered artificial neural network solves a given real-world decision task by learning from a large number of labeled examples, without being explicitly directed as to what to learn (LeCun et al., 2015; Kooi et al., 2017; Cao et al., 2018). For instance, in the aforementioned example of bank loans, the machine would be given a large number of actual client profiles, loan information that are appropriately annotated, or ‘labeled,’ as whether the given client defaulted on the given loan or not (Buyya et al., 2016; Crosman, 2016). Similarly, self-driving cars learn from a large number of suitably labeled pictures, videos and other driving-related data.

It is easy enough to intuit why learning (especially deep learning) and explainability are closely related. If a given task can be based on some type of straight-forward decision rule (e.g., if A, then B), then the task can be performed without resorting to training examples. Explainability is a moot issue in such cases, because the decision-maker (or the server, in the present context) need only cite the rule to explain his/her decision. On the other hand, when the underlying data are complex and variable enough, they tend to defy simple rule-based decisions, so that the task must be learned based on sufficiently large number of labeled examples. That is, absence of a readily specifiable decision rule is typically what makes learned decisions necessary in the first place. When deep learning is used to learn these decisions, explaining them becomes even harder, because deep learning often involves a very large number of parameters (millions or billions) that are organized purely for the efficiency of learning and not to be easily understood by humans. Thus, explainability is intricately related to deep learning, and vice versa.

There are notable parallels between decision-making by machine experts and by human experts. Many human experts also learn from labeled examples. For instance, an expert radiologist who learns to look for diagnostic patterns of breast cancer in mammograms cannot be explicitly taught exact rules as to what to look for. While radiological trainees are typically told where to look and what to look for (Homer, 1980; Nodine and Krupinski, 1998; Drew et al., 2013; Grimm et al., 2014), the underlying diagnostic image patterns are too abstract and variable, and the similarity between cancerous and non-cancerous image patterns are too subtle, for rule-based decision-making. Instead, the radiologist must learn from a sufficiently large number of labeled examples as to what constitutes possible cancer and what does not. But expert radiologists typically find it all but impossible to put into words, or explain, to their patients, insurance companies or even fellow experts, exactly how they arrived at the decision in precise enough terms so that another person can arrive at the same conclusion based on the same underlying data (Sevilla and Hegde, 2017).

In the context of biological systems, there are no broadly accepted terminology or definitions of explainability or related concepts. Therefore, we adopt in this study the following functional definitions, informed by the corresponding antecedent machine learning counterparts. We coined the term “weakly guided learning” to refer to a type of perceptual (or sensory) learning (Fahle and Tomaso, 2002), in which the subject learns to perform statistical decision-making using implicitly or explicitly labeled examples without being told what to learn or how to decide. For example, rather than being instructed that “flowers of the apple tree have five petals,” the learner is simply presented with examples of flowers, some labeled “apple” or “not apple,” and needs to learn without additional guidance. Thus, weak guidance is provided in the form of labels, but no additional guidance is given. In this sense, weakly guided learning is distinct from other forms of biological learning, or the machine learning concepts of both supervised and unsupervised learning. Similarly, we define explainability as the extent to which the criteria underlying a given decision can be stated in explicit, objective terms so that another observer is likely to arrive at the same decision by applying the same decision-making methodology in the explanation to the same underlying data. Note that by this operational definition, the notion of explainability of an explanation is comparable to the notion of implementability (e.g., of guidelines) in fields such as medicine (Shiffman et al., 2005).

Given the underlying similarity of decision-making among highly trained human experts in variety of fields, it is likely that explainability is an issue of great import in a variety of fields involving human experts, not just medicine. For one thing, it is abundantly clear that weakly guided learning, sometimes referred to as implicit learning in the cognitive psychological literature (Seger, 1994; Forkstam and Petersson, 2005; Jiang and Leung, 2005; Bart et al., 2008; Hegdé et al., 2008; Chen and Hegdé, 2010; Kromrey et al., 2010; Gao and Wilson, 2014), is a common mode of human learning. However, while explainability of machine decisions have recently received some attention (de Visser, 2012; Goldstein et al., 2015; Gunning, 2016; Lipton, 2016; Ribeiro et al., 2016a,b; Van Belle et al., 2016; Doshi-Velez and Kim, 2017; Fernandes et al., 2017; Ferrante, 2017), explainability of human decisions has not been systematically studied at all. Obviously, our lack of understanding represents a major barrier to progress in our understanding of human decision-making.

The goal of the present study is to take the first necessary steps to help overcome this barrier. To this end, we will use human categorical decision-making as an illustrative case and utilize a set of rigorous, machine learning-inspired methodological tools we have previously described (Bart et al., 2008; Hegdé et al., 2008; Kromrey et al., 2010; Hauffen et al., 2012). For clarity and convenience, we designate the original decision-maker as the ‘server’ and the subjects who subsequently utilize the servers’ decisions and explanations as the ‘clients.’ Note that, in principle, servers and/or clients can be either experts or naïve subjects themselves. Using this framework, we will illustrate some first-order principles of human explainability, and the methodological issues, that underlie human decision-making.

## Materials and Methods

### Subjects

A total of 13 subjects participated in this study. All were adult volunteers with normal or corrected-to-normal vision. All subjects gave written informed consent prior to participating in the study. All procedures related to study subjects were approved in advance by the Institutional Review Board (IRB) of Augusta University, where the experiments were carried out.

### Stimuli

**FIGURE 1:**
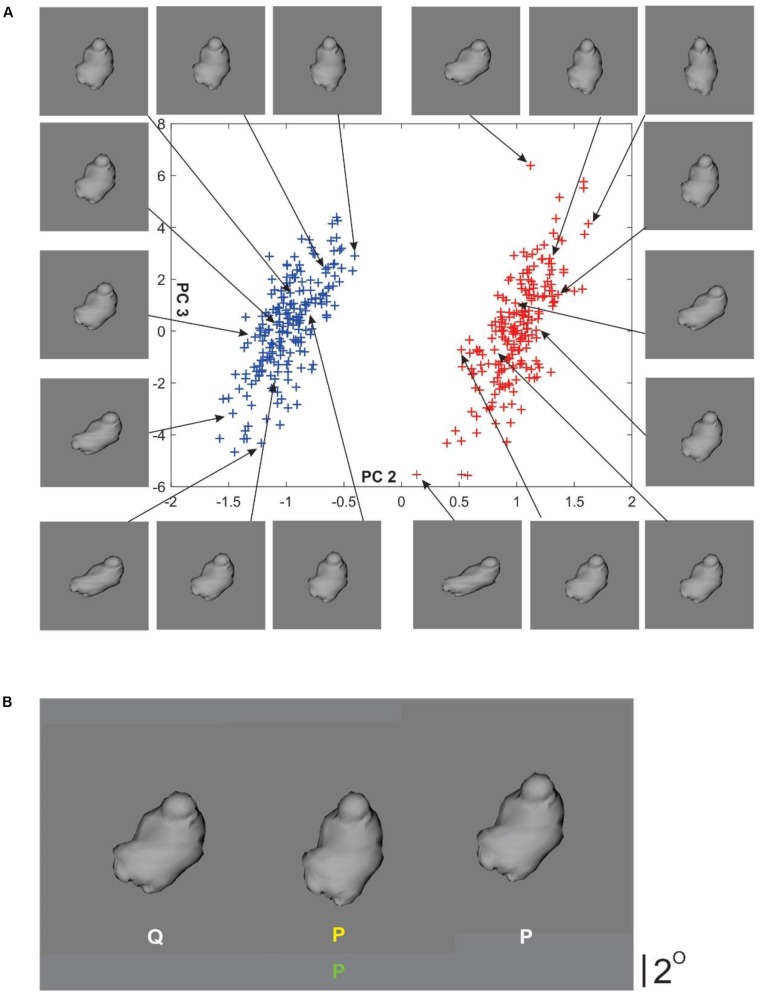
Stimuli and trial paradigm. **(A)** Digital embryo categories P and Q used in this study (*blue* and *red symbols*, respectively, in the scatterplot). Each plotting symbol in the scatterplot represents an individual embryo. The *x* coordinate of each given embryo represents the loading of principal component 2 (PC2) in the eigenspace (or shape space) of this set of embryos. Similarly, the *y* coordinate of the given embryo represents the loading for PC3. Thus, the 3D shape of a given embryo is fully specified by specifying its coordinates, and the categories are fully specified by specifying the mean (i.e., 2D center) and the spread (i.e., 2D variance) of the 2D Gaussian distribution. For visual clarity, the *x*- and the *y*-axes are plotted to different scales. *Arrows* denote individual embryos from each category selected to help illustrate the variations in shape characteristics specified by either PC. See text for details. **(B)** Task paradigm during the training phase. During each trial, subjects performed a binary categorization task. This panel illustrates a typical trial screen, in which the subjects were presented with three embryos. The *left* and *right* embryos are sample embryos randomly drawn from the category denoted by the appropriate category label (*white letters* beneath the embryos). The *center* *embryo* is the query (or test) embryo. The subject’s task was to classify the center embryo into either category. Subjects were allowed to view the embryos and toggle their categorical report (*yellow letter* beneath the center embryo) *ad libitum*, and were required to finalize the reported category by pressing a separate key. After the subjects finalized their report, they received feedback in the form of the actual category label of the query embryo (i.e., center embryo), which appeared beneath the reported category label. The color of the feedback label denoted whether the subject’s response was correct, so that the feedback label was *green* or *red*, depending on whether the response was correct (shown in this figure) or incorrect (not shown), respectively. The trial paradigm during the testing phase was identical (not shown), except that no feedback was provided. See text for details.

Stimuli consisted of two categories of novel, naturalistic 3-D objects called ‘digital embryos.’ We have previously outlined the usefulness of these objects as rigorous methodological tools for studies involving recognition and learning of objects and object categories (Bart et al., 2008; Hegdé et al., 2008; Kromrey et al., 2010; Hauffen et al., 2012), which we will summarize here briefly. First, this methodology allows the user to precisely specify all aspects of object shape and category properties (**Figure 1A**), so that the underlying categorical decision can be specified and analyzed in precise, machine learning terms (Bart et al., 2008; Hegdé et al., 2008; Kromrey et al., 2010; Hauffen et al., 2012). This is especially useful in the present study, which aims to ‘port’ the machine learning concept of explainability to cognitive science. Second, this methodology also allows the experimenter to systematically *manipulate* all aspects of the underlying categorization task, including but not limited to object appearance and task difficulty. We took advantage of this to ensure that the categorization task was, on the one hand, difficult enough so that it cannot be performed above chance levels without first acquiring the requisite perceptual expertise. We also ensured, on the other hand, that the task was easy enough that sufficiently large number of healthy but naïve subjects could learn the task to criterion within a few hundred trials spread over several sessions (Bart et al., 2008; Hegdé et al., 2008). Third, as we have also shown before, both humans and monkeys can learn categories using a form of weakly guided learning (Bart et al., 2008; Hegdé et al., 2008; Kromrey et al., 2010). This is especially useful in the context of the present study because, as noted above, explainability issues are particularly prominent in tasks learned in a weakly guided manner.

We created two categories of digital embryos using principal components analysis (PCA) of 400 randomly generated embryos each with 1474 vertices, using the methodology described by us previously (Hauffen et al., 2012) (also see **Figure 1A**). Briefly, principal components (PCs) represent the eigenvectors of this 400 × 1474 matrix. To create each given category, we used a 2-D Gaussian point process whose mean and variance were specified as 2-D coordinates of a PC space whose two axes were given by PCs 2 and 3, i.e., eigenvectors with the second and third highest eigenvalues. These two PCs were selected because, by visual examination, they produced subtler shapes. Using pilot experiments, we adjusted the *F* ratio of the two categories (i.e., between-category variance/within-category variance) so that, on the one hand, the categorization task could not be performed above chance levels without learning the categories, and, on the other, the task was easy enough that the subjects were able to learn it eventually. Individual embryos were rendered using programs custom-written using the OpenGL graphics toolkit^1^ and written to disk as standard BMP images.

### General Procedures

Experiments were controlled and the data was collected using scripts custom-written in the Presentation scripting language^2^. All experiments were carried out using randomized blocks of 40 trials each. Individual trials, trial blocks and experimental sessions were self-paced by the subject for maximum comfort.

Subjects typically carried out multiple blocks during each session, and participated in multiple sessions spread over several days and weeks (see section “Results”). At the beginning of each session, subjects carried out practice trials to help ensure that they were thoroughly familiar with the task paradigm or, if the subjects were continuing with a task paradigm they already knew, to help ensure that they were adequately ‘warmed up.’ Data from practice trials were discarded.

### Experiment 1: Learning-Dependent Changes in Categorization Performance

This experiment was carried out in three successive phases as described below. Each phase used a slight variation of the trial paradigm that we have described before (Bart et al., 2008; Hegdé et al., 2008; Kromrey et al., 2010).

#### Pre-training Test Phase: Measuring the Baseline Performance of the Servers

This phase consisted of two blocks of trials. Each trial began with the presentation of a small ‘+’ sign (0.4° visual angle) on a neutral gray background (not shown). When the subject indicated readiness by pressing a designated key, the ‘+’ sign was replaced by three digital embryos, each subtending about 6° (**Figure 1B**). The embryos on the left and right of center were sample embryos, each randomly drawn from the corresponding category P or Q, noted with the appropriate category label (*white letters* in **Figure 1B**). The center embryo was the query (or test) embryo, drawn randomly from category P or Q, depending on the trial. The spatial location of each embryo was jittered by up to 0.8°, so as to minimize the chance that the subject performed the task by pixelwise comparison of the embryos. The left vs. right location of the sample embryos from a given category, and the category from which the query embryo was drawn, were randomized from one trial to the next.

##### Task

Subjects were asked to view the embryos *ad libitum* and indicate, using a toggle key, which of the two categories the query embryo was drawn from. The subject’s response appeared as a *yellow letter* beneath the query embryo (**Figure 1B**). After the subjects pressed a separate key to finalize their categorical response, the next trial started. That is, subjects received no feedback after their response.

#### Training Phase: Weakly Guided Learning of Categories by Servers

This phase was identical to the pre-training test phase above, except as follows: first, during each trial, after the subjects finalized their categorical response, they received feedback in the form of the actual category label of the query embryo (*green letter at bottom center*, **Figure 1B**). Subjects were allowed to re-examine the stimuli *ad libitum* in view of the feedback. Note that this task paradigm fully meets the aforementioned operational definition of weakly guided learning, because subjects are not told what to learn, and had to learn the categories solely from labeled examples.

Second, subjects carried out as many trial blocks as necessary until they were trained to criterion. Subjects were considered trained to criterion when they performed at least three consecutive blocks at a *d*′ of ≥1.68 [which, for Gaussian data, corresponds to hit- and false alarm rates of about ≥80% and ≤20%, respectively (Green and John, 1966; Macmillan and Creelman, 2005)]. As an empirical matter, however, most servers performed better than the minimum criterion (see section “Results”).

#### Post-training Test Phase: Measuring the Performance in the Absence of Feedback

The post-training test phase was identical to the pre-training test phase in all respects.

### Experiment 2: Explainability of Server’s Categorical Decisions

This experiment was identical to pre-training and post-training test phases of Experiment 1 above, except as follows. First, only expert subjects, who had been trained to criterion in Experiment 1, participated in this experiment. For convenience, we will refer to the subjects in this experiment as servers, because they ‘served up’ the explanations for subsequent use by other subjects, or clients.

Second, during this experiment, after finalizing the categorical response during a given trial, subjects did not receive feedback. Instead, they were required to provide a written explanation of unlimited length that accounted for their decision as thoroughly as possible. They were also informed that other subjects will scrutinize and rate their explanations for both the extent to which it is semantically understandable (understandability) and the extent to which the explanation accounts for the decision (explainability). They were told that a good explanation is one which should enable another subject to perform the task accurately based solely on the explanation, without having encountered the categories beforehand or knowing the actual decision. That is, we emphasized to the servers that the explanations should be as self-explanatory as possible, i.e., they should be stand-alone in nature.

Our pilot experiments revealed the potential for subject fatigue (and the associated confounds) resulting from the server having to type similar explanations multiple times (data not shown). Therefore, in the actual experiments, we allowed servers to re-use, with or without additional editing, one of their previous explanations.

Third, after the subjects finalized their explanation, they were required to rate the explainability of their own explanation, using an on-screen sliding scale of 0 (the explanation does not account for the decision at all) to 100 (the explanation fully accounts for the decision). We will refer to this rating as the subjective explainability rating of the server (SER_S_).

### Experiment 3: Evaluation of Server’s Decisions and Explanations by Clients

This experiment was identical to Experiment 2 above, except as follows. First, both naïve and expert subjects participated in this study (as opposed to Experiment 2, in which all subjects were trained experts). For convenience, we will refer to the subjects in this experiment as clients, since they utilized the information provided by the servers.

Second, the clients were either naïve or were experts, depending on the particular variation of this experiment (see section “Results”). Also depending on the given variation of this experiment, clients were told that the server data came from either naïve servers or expert servers.

Third, the clients did not have to generate explanations of their own. Instead, during each trial, a categorical decision and/or explanation for the decision from a server were provided to the clients on the computer’s screen below query and sample embryos. All the stimuli provided to the client during a given trial were drawn from a single corresponding trial from Experiment 2 (i.e., the same set of individual embryos that the given server based his/her decision and explanation in Experiment 2 were also presented to the client in this experiment without any shuffling). Based on this information and on the embryos, the subjects had to categorize the query embryo.

Fourth, after the clients finalized their categorical decision, they had to rate the given explanation as to the extent to which it was semantically understandable (‘objective’ understandability rating, or OUR), and as to the extent to which it accounted for the given decision (‘objective’ explainability rating, or OER).

### Experiment 4: Characterizing How Clients Evaluated the Server Data

This experiment was identical to Experiment 3 above, except we varied the quality of information provided to the subjects. In one variation of the experiment (Experiment 4A), the given server’s categorical decisions during various trials were randomly shuffled with respect to the same server’s explanations. The aim of this experiment was to determine the extent to which the clients jointly evaluated the given server-decision and server-explanation.

In the second variation of this experiment (Experiment 4B), we further scrambled the server data (i.e., in addition to the scrambling in Experiment 4A) to randomly shuffle the category *labels* of the sample stimuli with respect to the stimuli themselves.

In neither Experiment 4A nor 4B were the subjects told that the server data were being scrambled in any way. This may have misled subjects to incorrectly believe at the beginning of the experiment that the data provided were reliable (as in previous experiments), and may therefore have caused confusion–for example, when the server’s explanation clearly suggests one category, but a (scrambled) expert’s label suggests a different category. Eventually, this may have also caused subjects to doubt the accuracy of the experimenters’ instructions. To minimize the possible influence of these factors, both Experiments 4A and 4B were only performed after a subject has already completed Experiments 1–3. Thus, any perceptions of unreliability of server data formed as a direct result of participating in Experiments 4A and 4B could not have influenced the results of Experiments 1–3. Note that misleading subjects in this manner may have had implications outside the immediate Experiments 1–4 performed in this paper, although the prevalence and effects of that are disputed. Some authors (e.g., Ortmann and Hertwig, 1998) state that it may contaminate the participant pool in the long run, while others (e.g., Christensen, 1988, p. 668) note “that research participants do not perceive that they are harmed and do not seem to mind being misled.”.

### Data Analysis

Data were analyzed using software custom-written in R (R Core Team, 2015) or Matlab (Natick, MA, United States). Subjects’ categorization performance was measured using the standard signal detection theoretic measure *d*′ (Green and John, 1966; Macmillan and Creelman, 2005). For the purposes of calculating the *d*′ values shown in this report, we arbitrarily designated ‘hits’ and ‘false alarms’ as correct and incorrect classification, respectively, of category P embryos. Using the opposite designation, where hits and false alarms were defined as the correct and incorrect classification, respectively, of category Q, yielded qualitatively similar results (not shown). Where appropriate, results of statistical tests were corrected for multiple comparisons using Tukey’s Honestly Significant Difference Test (Toothaker, 1993; Crawley, 2002; Hothorn and Brian, 2014).

## Results

### Weakly Guided Learning of the Categorization Task

**FIGURE 2:**
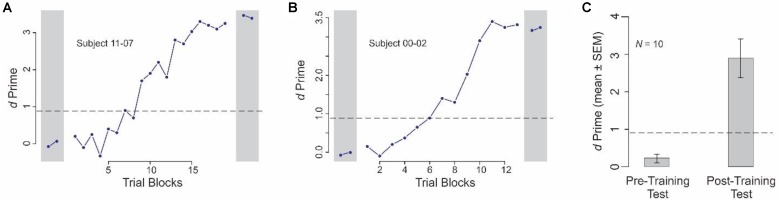
Learning-dependent improvement in categorization performance in Experiment 1. This figure shows the data from the different phases of Experiment 1. **(A,B)** Categorization performance of two individual subjects during pre-training test and post-training test phases (*gray rectangles* on *left* and *right*, respectively), and the training phase (intervening blocks). Note that the data are plotted to different *x*- and *y*- scales for the two subjects. **(C)** Average performance across all subjects before and after learning (pre-training test and post-training test phases, respectively). In each panel, the *dashed line* denotes the level at which the performance is statistically significant at *p* < 0.05, subject to Gaussian assumptions. Throughout this report, error bars denote SEMs, calculated across all relevant blocks and subjects. Error bars are not shown in **(A,B)** of this figure, because each data point therein represents the performance during a single given block.

All servers, and a subset of the clients who served as expert clients, were trained in the categorization task using Experiment 1 (see section “Materials and Methods”). **Figures 2A,B** show the categorization performance of two individual subjects before, during and after the weakly guided learning of the categories (see legend for details). Note that the number of trial blocks (of 40 trials each, see section “Materials and Methods”) needed to reach the criterion level of performance differed slightly between the subjects. On an average, subjects needed 19 trial blocks (range, 14–27 blocks; median, 19 blocks; SEM, 1.52; data not shown), spread over an average of 4.8 sessions (range, 3–7 sessions), or average of 15.6 calendar days (range, 8–24 calendar days).

Across all subjects, the performance was indistinguishable from random before training (**Figure 2C**, *left*; *p* > 0.05). After learning the categories, subjects were able to perform the task at highly significant levels (**Figure 2C**, *right*; *p* < 0.05). Moreover, the after-training performance was statistically significant in each individual subject (*p* < 0.05; not shown).

### Key Characteristics of Explanations Provided by Servers

Servers trained to criterion performed Experiment 2, in which they not only classified a given embryo during each given trial, but also provided a written explanation for their classification decision, and provided a rating, SER_S_, of the extent to which the given explanation accounted for the given decision (see section “Materials and Methods” for details). Representative explanations that elicited relatively high levels of performance in subsequent experiments with naïve clients are summarized in **Table 1A**. Similarly, representative explanations that elicited relatively poor performance are shown in **Table 1B**.

**Table 1A:** Examples of effective explanations: selected explanations by expert servers that led to relatively high classification performance by naïve clients^∗^.

Line #	Query embryo		Servers					Clients		
	Image	Category	Server ID	Reported category	Explanation^¶^	% Correct^§^	SER_S_	% Correct^§^	Mean OUR_NC^†^_	Mean OER_NC^†^_
1	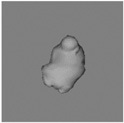	Q	11-58	Q	Triangle at the end of the neck vein is shadowy for Q	100	52	77.7	100	57.8
2	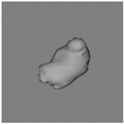	Q	11-07	Q	Shadow on right side of neck is dark for P and light for Q.	100	98	58.9	100	81.2
3	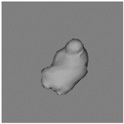	P	00-17	P	Shading on right side P’s neck is not very sharp nor dark.	100	100	70.0	90.9	70.2
4	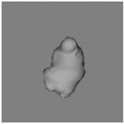	P	00-02	P	Shading of P is lighter and less sharp on right side of neck than shading on Q, more similar to P	100	100	58.2	69.5	66.0
5	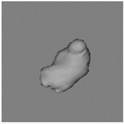	P	11-58	P	P has lighter shading around neck and Q had darker shading around neck	100	66.2	67.7	100	75.5

*^∗^All data in this table were collected from naïve clients who were informed that the explanations came from expert servers. See text for additional details. ^¶^ Each explanation shown in this column was provided by a single server because, as an empirical matter, different servers never provided mutually identical explanations. ^†^Data shown in the sub-columns of this column are aggregate data pooled across multiple trials and multiple clients. ^§^ Performance is reported here as percent of correct trials rather than d′ value, because in many cases there were zero trials corresponding to false alarms (in case of servers) or hits (in case of clients), which is problematic in calculating d′ (Green and John, 1966; Macmillan and Creelman, 2005).*

**Table 1B:** Selected examples of ineffective explanations: explanations by expert servers that led to relatively low classification performance by naïve clients^∗^.

Line #	Query embryo		Servers					Clients		
	Image	Category	Server ID	Reported category	Explanation^¶^	% Correct^§^	SER_S_	% Correct^§^	Mean OUR_NC^†^_	Mean OER_NC^†^_
1	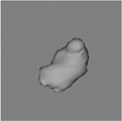	P	11-16	P	P has light and straight nerve tail	100	93	0	91.3	6.9
2	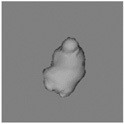	P	11-58	P	Q has harsher shading. Q has harsher shading than P	100	100	0	99.2	11.2
3	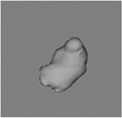	P	00-17	Q	Top right groove in P matches in length but not fully in shape	0	91	0	89.1	23.5
4	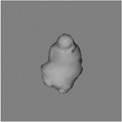	Q	11-16	P	P is smooth, dark and curvy	0	84	0	99.4	17.2
5	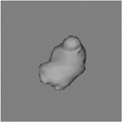	Q	00-02	P	Shading is pretty mild, more similar to P than Q	0	99	2.5	96.6	2.4

*^∗^All data in this table were collected from naïve clients who were informed that the explanations came from expert servers. See text for additional details. ^¶^ Each explanation shown in this column was provided by a single server because, as an empirical matter, different servers never provided mutually identical explanations. ^†^Data shown in the sub-columns of this column are aggregate data pooled across multiple trials and multiple clients. ^§^ Performance is reported here as percent of correct trials rather than d′ value, because in many cases there were zero trials corresponding to false alarms (in case of servers) or hits (in case of clients), which is problematic in calculating d′ (Green and John, 1966; Macmillan and Creelman, 2005).*

The explanations shown in these tables help illustrate some of the salient empirical properties of the servers’ explanations at large. First, even though all servers were highly trained experts in the task, they provided effective explanations in some trials and ineffective explanations in some others (see, e.g., explanations provided by Server #11–58 in both tables). This suggests not only that the ability to provide effective explanations was not limited to any individual server, and there was considerable within- and between-server variability in this regard, a fact confirmed by 1-way ANOVA of the performance elicited by the clients (*p* < 0.05 for server factor). Second, servers tended to rate their explanations well regardless of their own performance (e.g., SER_S_ values on lines 3 through 5 in **Table 1B**), as confirmed a 2-way ANCOVA (SER_S_ values × servers; *p* > 0.05 for SER_S_ values). This suggests that servers tended to overestimate the efficacy of their explanations. Finally, explanations that pointed to a spatially localized region of the image (e.g., lines 1–5 in **Table 1A**) tended to be more effective than explanations that referred to global features (e.g., lines 2, 4, and 5 in **Table 1B**). This is important, because it suggests that one potential strategy for improving the efficacy of explanations in this task is to train the servers to refer to specific, localized regions of the object. We will revisit this notion in the section “Discussion.”

### Explanations Can Enhance Clients’ Performance

**FIGURE 3:**
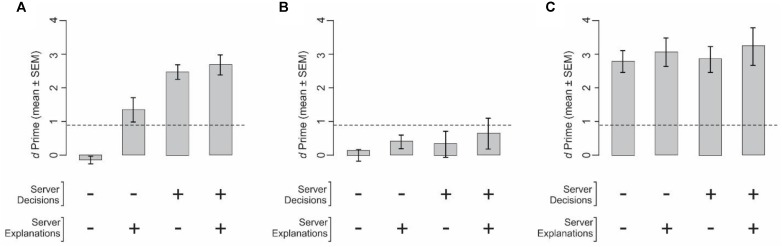
Performance of clients when provided with the servers’ decisions, explanations, both, or neither (Experiment 3). In this figure and **Figure 5**, the ‘+ sign means that given piece of information was provided, ‘–’ means it was not. **(A,B)** Performance of naïve clients (*N* = 4). **(C)** Performance of expert clients (*N* = 3). The underlying server data were identical in all three panels, and were obtained from Experiment 2. **(A,C)** Clients were told that the server data came from expert servers. **(B)** Clients were told that the server data came from naïve servers. In each panel, the *dashed line* denotes the level at which the performance is statistically significant at *p* < 0.05, subject to Gaussian assumptions. The performance of expert clients when they were told that the server data came from naïve servers, the client was performance was somewhat lower, but not significantly so (not shown). See text for details.

We measured the performance of the clients using expert servers’ decisions, explanations, both or neither (Experiment 3; see section “Materials and Methods”). When clients were naïve and were provided neither the server’s decision nor the server’s explanation during a given trial, they performed as chance levels, as expected (**Figure 3A**, *far left bar*). When naïve clients were provided the expert server’s explanation for the decision but not the decision itself, the performance did improve, and reached significant levels (*second bar from left* in **Figure 3A**; *p* < 0.05). Interestingly, naïve clients performed even better when they were provided only the decisions, but not the explanations, of the servers (*third bar from left*), suggesting that when naïve clients had access to the decisions of servers that they understood to be experts, they may have simply followed the expert opinions. The fact that providing additional information in the form of explanations for the decisions did not further improve the performance (*far right bar* in **Figure 3A**) lends support to the notion that when expert opinions were available, clients simply conformed to the expert opinions, and did not make the extra effort it arguably takes to utilize the explanations.

The above results raise the possibility that naïve clients attach considerable importance to the perceived expertise of the servers. To test this possibility, we repeated this experiment in the same naïve clients and using the same underlying server data, except that the clients were told that the data came from naïve servers. In this experiment, clients reverted to their chance-level performance, regardless of whether or not they had access to server decisions, explanations, or both (*p* > 0.05; **Figure 3B**). It also suggests that clients may use different decision strategies based on the type of server data (decisions, explanations, or both) and/or the perceived level of server expertise.

When the clients were experts themselves, their performance levels were not significantly affected by information from the servers (one-way ANOVA, *p* > 0.05; **Figure 3C**). This result, however, may be at least in part because the clients’ performances were already near asymptotic levels (i.e., at performance ceiling), and cannot be solely because that the clients ignored the server data. These results are also consistent with our findings when expert clients were provided the same underlying server data but were told the data came from naïve servers (*p* > 0.05; data not shown).

### Explainability Is Different Than Understandability

It is evident from **Tables 1A,B** above that clients often rated the explainability of an explanation poorly even when they semantically understood the explanation, and *vice versa* (*cf*. OUR_NC_ and OER_NC_ values). This helps underscore a potentially important principle that while providing understandable explanations is evidently necessary, it is not sufficient for generating effective explanations.

**FIGURE 4:**
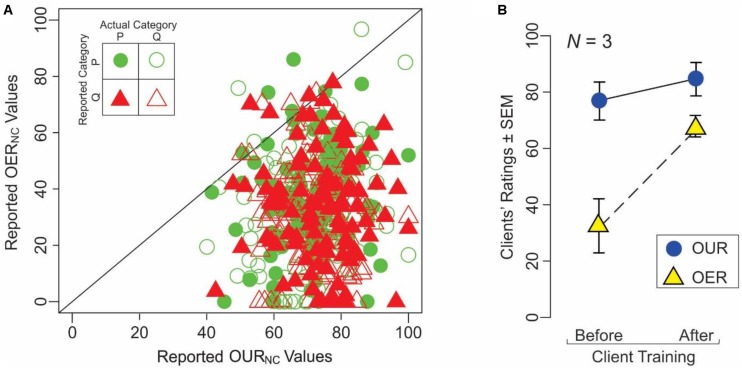
Understandability of explanations is differentiable from their explainability. **(A)** This panel summarizes a key result from Experiment 3, in which naïve clients (*N* = 5) were given the explanations provided by expert servers during individual trials, along with the stimuli on which the servers based their decision, but not the decision itself. The clients rated the explanations with respect to their understandability and explainability (OUR_S_ and OER_S_, respectively). The OUR_S_ and OER_S_ values are plotted in this figure against each other. Each *plotting symbol* represents a single pair of ratings of the client during a single trial, broken down according to the outcome of the trial as denoted in the legend (*inset* at upper left). The *diagonal* represents the line of equality between the two indices. **(B)** Learning-dependent changes in the understandability ratings (*blue circles*) and explainability ratings (*yellow circles*). See text for details.

To help examine this principle more quantitatively, we compared OUR_NC_ vs. OER_NC_ values from individual trials of naïve subjects in Experiment 3 (**Figure 4A**). Across all clients, OUR_NC_ values tended to be significantly higher than the OER_NC_ values (paired *t*-test, *p* < 0.05). This was true regardless of the outcome of the individual trial (see *inset* in **Figure 4A**; ANCOVA, *p* > 0.05). Moreover, OUR_NC_ values were uncorrelated with the OER_NC_ values across all clients (*r* = 0.07, *df* = 432, *p* > 0.05). Together, these results indicate that understandability of an explanation is different from its explainability.

The above results from naïve clients raise the possibility that at least part of the reason why naïve clients performed poorly using expert servers’ explanations is that the clients did not have the expertise to fully grasp what the explanations were referring to. That is, it is possible that it takes an expert to fully understand an expert.

If this is true, training a client in the task should improve the perceived explainability of the explanations. To test this hypothesis, we compared the OUR and OER values from three of the clients who participated in Experiment 3 before and after learning, i.e., as naïve and expert clients, respectively. Indeed, these clients showed a statistically significant, training-dependent improvement in OER values (*yellow triangles* in **Figure 4B**; paired *t*-test, *p* < 0.05). However, OUR values showed no significant training-dependent changes (*blue circles*; paired *t-*test, *p* > 0.05). These observations and the aforementioned fact that expert clients tended to perform the task at highly significant levels using the same underlying explanations (**Figure 3C**) are mutually consistent with each other, and suggest that expertise with the underlying decisions does indeed make it easier to enhance their explainability.

These findings are important, for two main reasons. First, they indicate the improvement in explainability is not attributable to an improvement in the understandability of the explanation at the semantic level. Second, explainability of a decision depends, among other things, on the level of expertise of the client. That is, explainability of decisions can be improved, at least in part, by training the clients appropriately. We will revisit this notion in the Section “Discussion” below.

### Perceived Expertise of the Servers Affects Clients’ Ratings of the Server Data

A line of social psychology studies pioneered by Solomon Asch and others has revealed a class of effects, often referred to as the Asch Conformity Effect, in which subjects attach much higher value to a set of data (such as, say, external opinions) if the subjects value the source of the data in some respect (Asch, 1956; Schulman, 1967; Mertesdorf et al., 1969; Stamps and Teevan, 1974; Bond and Smith, 1996; Walker and Andrade, 1996). We have described a variant of this effect above (**Figures 3A,B**) in which the same underlying server data led to better performance if the clients perceived the data to come from expert servers.

**FIGURE 5:**
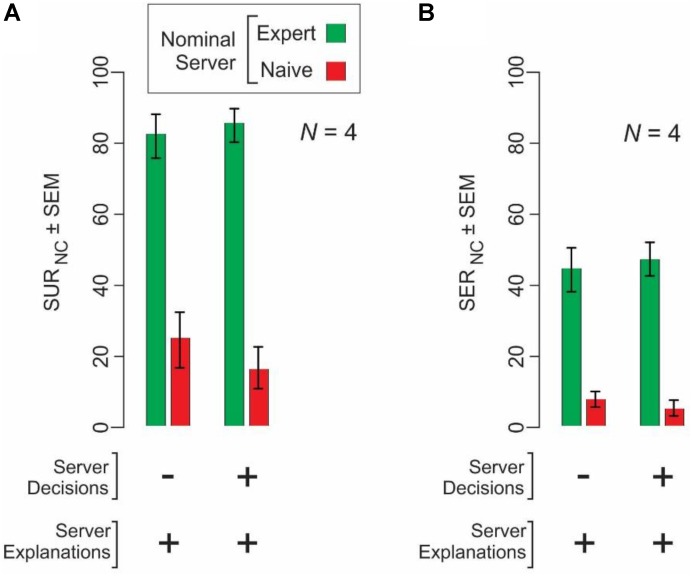
Naïve clients (*N* = 4) show conformity to the explanations of expert servers, but not naïve servers. Naïve clients rated the understandability **(A)** and explainability **(B)** of the same underlying server data under two different conditions, wherein they were told that the server data were from expert servers (*green bars*) or from naïve servers (*red bars*).

We directly measured whether the naïve clients’ perception of the level of the server’s expertise also affected their perception of the extent to which the server data are understandable and explainable. We found that this was indeed the case (**Figure 5**). OUR_NC_ and OER_NC_ values were significantly higher for server explanation from nominal experts than for the same explanations when they were perceived to come from naïve servers (2-way ANOVA, server expertise level × server data type; *p* < 0.05 for server expertise level and server data type, and *p* > 0.05 for interaction). Not surprisingly, comparable conformity effects were not evident at statistically significant levels in expert clients (*N* = 2; *p* > 0.05; data not shown).

Taken together with the results in **Figure 3**, these results demonstrate a potential variant of the Asch Conformity Effect in the context of decision explainability.

### Clients’ Decision Strategy Depends on the Reliability of the Server Data

The aforementioned result that the performance of naïve clients remains about the same when the clients are provided server explanations in addition to server decisions (**Figure 3A**) raises the possibility that the clients simply ignore subject explanations when subject decision information is available, but not when it is not.

To help characterize the clients’ decision strategy, we scrambled the decisions with respect to the explanations, so that the server explanations and visual stimuli, but not the server decisions, remained identical to with those encountered or reported by the given server (Experiment 4A; see section “Materials and Methods”). We hypothesized that the clients’ explanations should deteriorate to the extent to which the client down-weights the server explanation, visual stimuli, or both.

**FIGURE 6:**
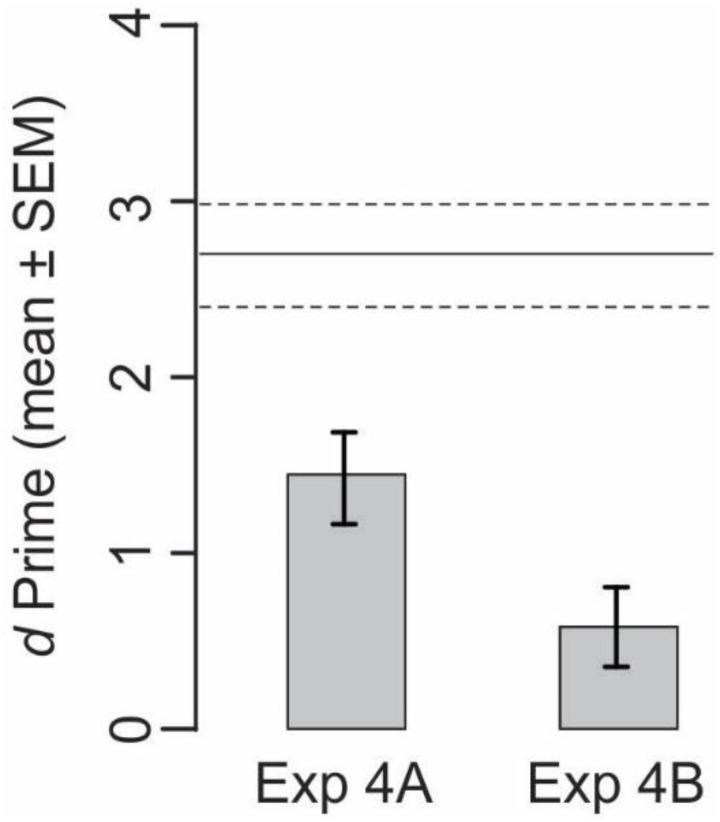
Performance of naïve clients when the data from nominal expert servers are scrambled. Performances from four subjects each in Experiments 4A and 4B are shown. See text for details. *Horizontal lines* denote the corresponding unscrambled server data from **Figure 3A**, far right bar (labeled “Server Decisions+, Server Explanations+), redrawn here for reference (mean, *solid line*; SEMs, *dashed lines*).

Two aspects of the results from this experiment are notable (**Figure 6**, *left bar*). First, the client performance based on scrambled server data significantly deteriorated compared to the performance elicited by unscrambled server data (*cf. horizontal lines* in **Figure 6**; *t*-test, *p* < 0.05), confirming the aforementioned result (**Figure 3**) that naïve subjects attach substantial weight to server decisions. Second, performance still remained at statistically significant levels (*t*-test, *p* < 0.05), suggesting that the clients were still utilizing the explanations, which remained unscrambled, and therefore reliable. Under this scenario, clients evaluate the server explanations in light of the visual stimuli in order to make the best decision possible in light of the available data.

To test this possibility, we carried out Experiment 4B, in which we further scrambled the server data (i.e., in addition to the scrambling in Experiment 4A) to randomly shuffle the category *labels* of the query stimuli with respect to the sample stimuli. We hypothesized that if the subjects use the aforementioned strategy of evaluating the explanation in light of visual evidence, the scrambling of server data in Experiment 4B should deteriorate the performance back to chance levels. This is indeed what we found (**Figure 6**, *right bar*). This suggests, albeit by no means proves, that the clients do indeed use a fairly rational strategy wherein they evaluate all available server data against each other before making their own categorical decision.

## Discussion

### Importance of Explaining Expert Decisions

When a human expert’s decision significantly affects other people, it is desirable to have an explanation for why the decision was made. Such explanations serve several purposes:

• They allow clients or patients to understand the rationale behind the expert’s decision, making it seem less like a sleight of hand and more like a principled, objective procedure. In a medical context, this may improve patient compliance, leading to improved outcomes.
• They allow the expert’s teammates to build a model of the expert’s behavior (for example, understanding the conditions under which the expert is particularly prone to errors or may require longer than average time to make a decision). This, in turn, can lead to improved trust between team members.
• They make it easier for others to review the expert’s work. For example, explanations can be useful for determining whether a decision subsequently determined to be incorrect was reasonable under the circumstances when it was made, or identifying conditions where the expert makes systematic errors for potential additional training.
• They make it easier for trainees undergoing expertise training (e.g., medical residents) to acquire the decision-making expertise.

Explanations are particularly useful when the underlying expertise was acquired through some form of weakly guided learning. In those cases, decisions made by the expert may seem almost “magical” to naïve users, since naïve users (and often the experts themselves) do not have an explicit understanding of how they are in fact made. This effect is significantly reduced when the expert performs the task by following an algorithm (such as when solving a quadratic equation via the quadratic formula method), because the individual steps can be readily traced by other users.

It is therefore desirable to study explainability of human expert decisions, particularly in the context of weakly guided learning. Of particular interest would be understanding what makes an explanation useful to its intended audience, how to improve the explainability of expert decisions, and the relationship between the explainability and the performance of the experts and their teammates and clients. The current study is a first step toward this goal.

We obtained several interesting results in this study. First, the experts’ explanations improved the categorization performance of naïve users, but this effect differs significantly between explanations. In particular, explanations that pointed to a spatially localized region of the image improved the user’s performance much better than explanations that referred to global features. This suggests that it may be possible to improve the explanations by encouraging experts to formulate them in ways that other users typically find most helpful. Note, however, that it must be done carefully, since altering the expert’s workflow in this manner has the potential to also affect the expert’s performance. For example, if global features provide more reliable information about the correct category label, then asking the expert to focus on local feature may reduce their accuracy and/or make explanations misleading (in that an explanation in terms of local features will not reflect the true process of arriving at the decision via global features). Further studies are necessary to understand the relationship between the explanations the expert produces and their performance in the main task.

Second, we found that neither experts themselves nor the naïve users were able to predict how useful a given explanation will be. Since the ultimate goal is to have useful explanations, the ability to evaluate them ahead of the actual task is desirable. It is therefore useful to investigate how this ability may be improved.

Third, we observed significant bias effects, where naïve subjects rated an explanation significantly higher when told it came from an expert user, compared to the rating of the same explanation when told it came from another non-expert, suggesting a variant of the Asch conformity effect. Again, this requires further study to allow for controlling or eliminating this bias.

### Limitations

Several limitations of the current study need to be pointed out. First, we chose to study explainability in the context of a visual task (namely, visual object categorization). The reason was that in this context explainability effects are often most apparent: the expert will “just see” the correct category, but will often struggle to explain how they see it. However, one important caveat to keep in mind is the distinction between the expert not knowing why they made a decision and the expert knowing but being unable to verbalize the reasons. This is particularly important since in our experiments we used novel “digital embryo” objects which the subjects do not have standard, accepted terminology to describe. Of course, sensory expertise can involve senses other than vision (e.g., auditory sense for music critics, or the senses of taste and smell for wine tasters). In addition, many expert decisions are not sensory, but cognitive (for example, decisions to invest in a company or underwrite a mortgage) or a combination thereof (for example, the decision to appraise a given house at a certain value). Studying such tasks is a subject of future work.

Second, it is possible that one or more of our results are idiosyncratic to the particular objects we used. For instance, prior semantic or perceptual knowledge of natural object categories is of little or no use in case of digital embryo objects – indeed, these are among these reasons why digital embryos are so useful in the research on weakly guided learning (Bart et al., 2008; Hegdé et al., 2008; Kromrey et al., 2010; Hauffen et al., 2012). Also by design, our images each contained a single untextured, grayscale object, rather than, say a colorful visual scene with multiple natural objects. Therefore, it is possible that principles of explainability for complex natural images, where knowledge plays a greater role, may be different.

Third, for practical reasons, our study used one of the simplest possible client–server scenario. For instance, unlike in the real world, our clients and servers did not interact with each other, nor did the servers have an opportunity to revise their explanations, e.g., based on the clients’ queries, perhaps in an iterative and interactive fashion. Whether or to what extent our results will generalize to such complex scenarios remains to be determined (also see below).

### Relation to Explainability in Machine Learning

The need for AI systems to explain their decisions in a manner understandable to the system’s human operators became apparent quite early in the history of AI (Shortliffe and Buchanan, 1975; Fagan et al., 1980). Modern research in XAI (explainable AI) covers several broad areas. Some approaches focus on generating human-understandable explanations for an AI system that has already been constructed (for example, a pre-trained deep network). This is challenging because AI systems often involve complicated math and millions of individual parameters. Therefore, simply dumping the system’s internal state would not facilitate understanding, and creative approaches are needed to present that state in a useful manner. Methods such as those of Bojarski et al. (2017), who highlight image locations where the feature maps of a deep network have highest activations, and Kulesza et al. (2015), where the underlying conditional probabilities of a Naïve Bayes model are explained through user-friendly terminology and visualization, fall under this category. Other approaches aim to adjust a given AI system specifically so as to improve the quality of these explanations. Examples of such methods include Abdollahi and Nasraoui (2016), who introduce a problem-specific “explainability” term into the objective function their system optimizes, and Ribeiro et al. (2016c), who derive an easily explainable local linear approximation to a potentially complex non-linear decision boundary. Some methods also attempt to incorporate feedback from the users into the AI system (e.g., Kulesza et al., 2015).

While some of the same classifications are applicable to research in human expert explainability, the focus of research in each area is somewhat different. For example, in machine learning, the underlying mechanism by which the system performs its task is known to system designers; the challenge is just explaining it succinctly and to non-expert users. In contrast, the mechanisms by which human experts perform their tasks are not always known to experts themselves or even to researchers. As another example, an AI system can always be safely adjusted to improve its explanations, because it is possible to roll it back to any desired state if needed. In contrast, any changes to an expert’s workflow must be carefully monitored, because training an expert to produce better explanations (or even simply asking the expert to produce any explanations) may affect their performance at the main task of making decisions in the first place.

### Future Directions

The results obtained here suggest several interesting directions for future work. For example, we found significant differences in clients’ ability to use different explanations. This suggests it may be possible to train servers to produce explanations of the more useful kind with the needs and abilities of the clientele in mind. One possibility of accomplishing this is through an iterative process, where the server receives feedback and possibly requests clarification from the clients. A countervailing consideration in this regard is the necessity to balance producing more useful explanations against the causing unintentional changes to the server’s performance in the main task.

We have also observed significant bias effects; therefore, researching ways to control or eliminate this bias is another possible direction for future research.

Finally, we note that in the current study, each trial involved presenting a client with a single explanation from a single server. Since different servers may use different techniques for solving the same task, it may be of interest to develop automated methods for combining explanations from multiple servers to improve their usefulness.

## Conclusion

We establish a methodology for performing research in explainability of human decisions, provide promising initial results, and outline directions for future research.

## Ethics Statement

This study was carried out in accordance with the recommendations of the Institutional Review Board (IRB) of Augusta University. The protocol was approved by the Institutional Review Board (IRB) of Augusta University. All subjects gave written informed consent in accordance with the Declaration of Helsinki.

## Author Contributions

JH conceived the idea, designed and performed the psychophysics experiments, analyzed the data, and wrote the paper. EB conceived the idea, designed experiments, consulted in analyzing data, and wrote the paper.

## Conflict of Interest Statement

The authors declare that the research was conducted in the absence of any commercial or financial relationships that could be construed as a potential conflict of interest.

## Footnotes

1 https://opengl.org/

2 http://www.neurobs.com/
